# Metabolomic Profile of the Fungus *Cryomyces antarcticus* Under Simulated Martian and Space Conditions as Support for Life-Detection Missions on Mars

**DOI:** 10.3389/fmicb.2022.749396

**Published:** 2022-05-12

**Authors:** Federica Gevi, Patrick Leo, Alessia Cassaro, Claudia Pacelli, Jean-Pierre Paul de Vera, Elke Rabbow, Anna Maria Timperio, Silvano Onofri

**Affiliations:** ^1^Department of Ecological and Biological Sciences (DEB), University of Tuscia, Viterbo, Italy; ^2^Department of Environmental Sciences, Informatics and Statistics, University Ca’ Foscari of Venice, Venice, Italy; ^3^Italian Space Agency (ASI), Rome, Italy; ^4^MUSC, German Aerospace Center (DLR), Space Operations and Astronaut Training, Cologne, Germany; ^5^German Aerospace Centre, Institute of Aerospace Medicine (DLR), Cologne, Germany

**Keywords:** extremophilic microorganism, LC-MS, metabolites, osmolytes, stress resistance, biosignature

## Abstract

The identification of traces of life beyond Earth (e.g., Mars, icy moons) is a challenging task because terrestrial chemical-based molecules may be destroyed by the harsh conditions experienced on extraterrestrial planetary surfaces. For this reason, studying the effects on biomolecules of extremophilic microorganisms through astrobiological ground-based space simulation experiments is significant to support the interpretation of the data that will be gained and collected during the ongoing and future space exploration missions. Here, the stability of the biomolecules of the cryptoendolithic black fungus *Cryomyces antarcticus*, grown on two Martian regolith analogues and on Antarctic sandstone, were analysed through a metabolomic approach, after its exposure to Science Verification Tests (SVTs) performed in the frame of the European Space Agency (ESA) Biology and Mars Experiment (BIOMEX) project. These tests are building a set of ground-based experiments performed before the space exposure aboard the International Space Station (ISS). The analysis aimed to investigate the effects of different mineral mixtures on fungal colonies and the stability of the biomolecules synthetised by the fungus under simulated Martian and space conditions. The identification of a specific group of molecules showing good stability after the treatments allow the creation of a molecular database that should support the analysis of future data sets that will be collected in the ongoing and next space exploration missions.

## Introduction

The detection and identification of potential traces of extant or extinct life on other planets are currently one of the main challenges that next space exploration missions must face (Parnell et al., 2007). To overcome this challenge, the discovery of an object, substance, or pattern whose origin specifically requires a biological agent, called biosignatures (Horneck et al., 2016; Carrier et al., 2020), is essential. The ongoing life-detection missions on Mars, Europa, Enceladus, and other planetary bodies will focus on those biological components that are necessary to sustain life as we know it. In this context, since life on Earth is the only example we know so far, it is crucial to study the stability and degradation of biological molecules synthesised by terrestrial microorganisms, to obtain a reference for the life-detection missions on other planetary bodies. Many terrestrial microorganisms are known as extremophiles, adapted to survive the harsh conditions of the inhospitable habitats on Earth. These extreme habitats are characterised by environmental conditions like those experienced on other planetary bodies of our solar system. Among these environments, the McMurdo Dry Valleys (Southern Victoria Land, Antarctica) is one of the closest terrestrial analogues of Mars, being the coldest hyper-arid desert on Earth (Friedmann, 1982; McKay and Friedmann, 1985; Friedmann et al., 1987; Aerts et al., 2014). In these regions, microorganisms are subjected to harsh surface conditions, such as desiccation, thaw/freeze cycles, and radiation. To find shelter against superficial extreme conditions, life evolved beneath the surface, colonizing the porous rocks (Friedmann, 1982). It is likely that similar life forms could also develop or have developed on extraterrestrial planets. Indeed, the present-day Martian surface is characterised by intense radiation, oxidizing conditions, concentrated evaporative salts, and extremely low water activity (Davila et al., 2010). However, new observations suggest a warmer and wetter environment within the first half-billion years after its formation, characterised by a significant amount of liquid water at the surface (Nisbet and Sleep, 2001; Zahnle et al., 2007). Conditions then were similar to those microbes that originated and evolved on young Earth (Vago et al., 2017). Current conditions of Martian surface (e.g., cold and dryness) may favour the preservation of traces of life, that may have formed under its more clement past history (McKay, 2007). Due to the inhospitable conditions of the present surface, potential habitat for extant life might exist in the subsurface, which hosts stable deposits of liquid water providing perfect niches for sustaining hypothetical living cells. For these reasons, Mars is one of the prime targets for the search for signs of life in our Solar System. Ongoing and future robotic exploration missions on Mars will drill the surface and subsurface [NASA’s Mars 2020 and the European Space Agency (ESA)–Roscosmos’s ExoMars 2022 missions, respectively] of the planet, to detect geomorphological-molecular remnants of past life in specific sites, supposed to had hosted water 3.5 billion years ago (Vago et al., 2017; Farley et al., 2020). However, the present-day Mars physicochemical conditions (e.g., radiation, oxidizing conditions, evaporative salts, and dryness) may alter the structure of molecules modifying their detectability by the instrumentations onboard the rovers (de Vera, 2019). The effects of these parameters on biosignatures detection should be deepened during ground-based experiments, to support the space exploration missions and better interpret the data that will be collected during the robotic operations.

In this context, ground-based experiments allow simulating of these parameters to prevent pitfalls and errors which may occur during the interpretation of data obtained *in situ* (e.g., on Mars). The results from the tests are essential to compare them with those obtained from the analyses performed by the instruments onboard the rovers. The current instrumentation within the payload’s rovers includes mass spectrometers, able to investigate the mass distributions of small organic compounds (Goesmann et al., 2017). In this context, metabolomics may assist in detecting quantifiable substances whose presence is indicative of extant or extinct life, since it allows to discriminate the intermediates and the end products of metabolism produced by living cells. The identification of biosignatures on other planetary bodies should begin with the identification of a pattern of molecules that are specific to metabolic pathways in similar environments on Earth. In this context, a metabolomic approach appears to be very useful to increase the database of possible molecular biomarkers (Seyler et al., 2020).

Here, the endurance of biomolecules of the extremotolerant fungus *Cryomyces antarcticus* after the exposure to the ground-based Science Verification Tests (SVTs), conducted in preparation for the flight mission EXPOSE-R2 as part of the Biology and Mars Experiment (BIOMEX) project (Rabbow et al., 2012, 2015) were analysed through a metabolomic approach. The ESA’s BIOMEX project aimed to investigate the (i) survivability of the selected extremophilic organisms and (ii) stability/degradation of biomolecules under simulated Martian and space conditions and into low Earth orbit (LEO; de Vera et al., 2012, 2019). Previous works reported the capability of *C. antarcticus* to tolerate different kinds of stressors (Onofri et al., 2007; Pacelli et al., 2017a,b,c, 2019, 2021; Aureli et al., 2020; Cassaro et al., 2021) and to resist in real space (Onofri et al., 2012) under simulated Martian conditions for 1.5 years in LEO (Onofri et al., 2015). Survivability studies conducted after SVTs treatments, reported a good metabolic activity and growth rate without significant DNA or ultrastructural damages (Pacelli et al., 2019). Recently metabolomics was successfully applied to Antarctic cryptoendolithic lichen-dominated communities, defining their adaptation strategies to survive in these harshest conditions. For this reason, metabolomics become a powerful tool to study life in extreme environments (Coleine et al., 2020; Fanelli et al., 2021). Here, this approach allows us to investigate (i) which fungal biomolecule maintains their stability after exposure to SVTs, and (ii) the effects of the interactions of two Martian regolith analogues on fungal growth as well as during dehydration processes.

## Materials and Methods

### Cultivation and Preparation of Dehydrated Fungal Strain

The Antarctic cryptoendolithic black fungus *C. antarcticus* MNA-CCFEE 515 was used as a test organism in this research. For the experiment, the fungal strain was kindly supplied by the Culture Collection of Fungi from Extreme Environments (CCFEE), kept in the section of the National Antarctic Museum (MNA) of the University of Tuscia (Viterbo, Italy). Three different cultivation media, containing different mineral mixtures, were prepared following specific protocols as described in Pacelli et al. (2017a).

Cultivation media were prepared with different mineral mixtures, previously crushed into fragments smaller than 1 mm (Böttger et al., 2012), and dry heat-sterilised for 4 h. In particular, 1.5 g of Antarctic sandstone [referred to as “original sandstone (OS) samples”], 1 g of Phyllosilicatic Mars Regolith Simulant analogue (P-MRS, referred to as “P-MRS samples”), which are simulating minerals formed during the Noachian epoch, and 1 g of Sulphatic Mars Regolith Simulant analogue (S-MRS, referred to as “S-MRS samples”), which are simulating the Hesperian/Amazonian epochs on Mars (Böttger et al., 2012) were added into a sterile falcon containing 40 ml of 2% malt extract agar (MEA). The content of each falcon was gently vortexed and transferred into different Petri dishes. The final product for all three-cultivation media was a solid mixture of MEA (approximately 0.5 cm high) containing uniformly powdered regolith analogues.

Fungal colonies (2,000 CFUs) were spread on each cultivation medium and incubated for 3 months at 15°C, obtaining superficial colonies that were well exposed outside the media. Lastly, each medium was cut creating disks of 12 mm diameter (named “pellets”) and then dehydrated overnight under sterile conditions. Each pellet was inserted into the wells of the exposure facility, EXPOSE-R2, according to the optimised protocol reported by Pacelli et al. (2017a).

### Ground-Based Simulations: Science Verification Tests

**FIGURE 1:**
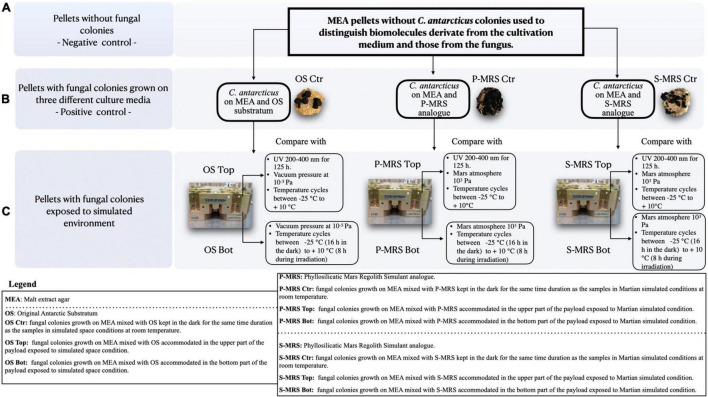
Experimental workflow. **(A)** pellets without fungal colonies (negative control) were compared to **(B)** pellets with fungal colonies grown on three different cultivation media (Positive control) containing three different mineral mixtures mixed with malt extract agar (MEA) to remove from the analysis all the metabolites derived from the growth medium (MEA). In **(C)** the same pellets from panel B were exposed to simulated Martian and space conditions in the upper and lower part of the payload.

The BIOMEX ground-based SVTs were carried out to confirm that all experiments and samples were accurately prepared for the space mission, and to achieve the highest possible scientific performance. As mission pre-flight tests, SVTs involved the exposure of all different extremophilic microorganisms foreseen for the respective mission to a combination of simulated Martian and space conditions within a copy of the flight EXPOSE-R2 facility, at the Planetary and Space Simulation facilities (PSI), Institute of Aerospace Medicine (German Aerospace Center, DLR, Koln, Germany). The accommodation setup of the samples within the wells was described in detail in de Vera et al. (2012). OS samples were exposed to space-like conditions, which include vacuum (10^–5^ Pa) exposure and temperature cycles within a range from −25°C (16 h in the dark) to + 10°C (8 h during irradiation; Figure 1). Among OS samples, the samples accommodated in the upper part of the hardware (referred to as “OS Top”) were exposed for 125 h to the polychromatic UV (200–400 nm) radiation produced by the solar simulator SOL 2000 (Dr. Hönle GmbH, Germany) with a dose of 5.5 × 10^5^ kJm^–2^ (Figure 1), whereas OS samples located below Top samples were shielded from radiation flux (referred to as “OS Bot”).

Furthermore, P-MRS and S-MRS samples were exposed only to Mars-like conditions, including temperatures cycles ranging from −25°C (16 h in the dark) to + 10°C (8 h during irradiation) and gases composition mimicking the Martian atmosphere composed of 95.55% CO_2_, 2.7% N_2_, 1.6% Ar, 0.15% O_2_, and *370 ppm H_2_O with a pressure of 10^3^ Pa (Figure 1). S-MRS and P-MRS samples (referred to as “S-MRS Top” and “P-MRS Top”) accommodated in the upper part of the layer, were exposed to the same radiation as described for OS Top samples. In the same way, “P-MRS and S-MRS Bot” were accommodated below the Top samples. Neutral density filters (0.1%) were used to attenuate radiation in all tests performed. The duration of exposures to simulated Martian and space conditions was 35 days which correspond to an exposure period of 12 months aboard the ISS, as estimated from previous EXPOSE data and simulations (Rabbow et al., 2012, 2015). Controls (referred to as “OS Ctr,” “P-MRS Ctr,” and “S-MRS Ctr”) were kept in the dark for the same time duration as the exposure samples at room temperature at DLR (Cologne).

MEA pellets without *C. antarcticus* colonies (referred to as “negative Ctr”; Figure 1), were used to distinguish biomolecules derivate from the cultivation medium and those from the fungus.

### Metabolomic Analysis

#### Metabolite Extraction

In this method, 1 g of each pellet (OS, P-MRS, S-MRS samples, and negative Ctr) was added to 1,000 μL of a chloroform/methanol/water (1:3:1 ratio) solvent mixture stored at −20°C. Briefly, samples were vortexed for 5 min and left on ice for 2 h for completed protein precipitation. The solutions were then centrifuged for 15 min at 15,000 × *g* and were dried to obtain visible pellets. Finally, the dried samples were re-suspended in 0.1 ml of water, 0.5% formic acid, and transferred to glass autosampler vials for liquid chromatography-tandem mass spectrometry (LC-MS) analysis.

### Ultrahigh-Performance Liquid Chromatography

In this method, 20 μL of extracted supernatant samples were injected into a UHPLC system (Ultimate 3000 Thermo, Germany) and run on a positive mode. Then, samples were loaded onto a Reprosil C18 column (2 mm × 150 mm, 2.5 μm-DrMaisch, Ammerbuch, Germany) for metabolite separation. Chromatographic separations were made at a column temperature of 30°C and a flow rate of 0.2 ml/min. For positive ion mode (+) MS analyses, a 0–100% linear gradient of solvent A (double-distilled H_2_O, 0.1% formic acid) to B (acetonitrile, 0.1% formic acid) was employed over 20 min, returning to 100% A in 2 min and holding solvent A for a 6-min post time hold. Acetonitrile, formic acid, and HPLC-grade water and standards (= 98% chemical purity) are all purchased from Sigma-Aldrich. The UHPLC system was coupled online with a Q Exactive mass spectrometer (Thermo Fischer, Germany) scanning in full MS mode (2 μ scans) at a resolution of 70,000 in the 67 to 1,000 m/z range, a target of 1,106 ions and a maximum ion injection time (IT) of 35 ms with 3.8 kV spray voltage, 40 sheath gas, and 25 auxiliary gas. The system was operated in positive ion mode. Source ionisation parameters were as follows: spray voltage, 3.8 kV; capillary temperature, 300°C; S-Lens level, 45. Calibration was performed before each analysis against positive or negative ion mode calibration mixes (Pierce, Thermo Fisher, Rockford, IL, United States) to ensure the error of the intact mass within the sub-ppm range. Metabolite assignments were performed using computer software (Maven, 18 Princeton, NJ, United States), upon conversion of raw files into a.mzXML format using MassMatrix (Cleveland, OH, United States). Analysis of each sample was performed in triplicate and a *p-*value of 0.01 was used for all abundance comparisons between sets of triplicates.

### Data Elaboration and Statistical Analysis

Replicates were exported as mzXML files and processed through MAVEN 5.2. MS chromatograms were created for peak alignment, matching, and comparison of parent and fragment ions with tentative metabolite identification (within a 2-p.p.m. mass-deviation range between the observed and expected results against an imported KEGG database). For multivariate analysis, data of all experiments were merged into a matrix table. Variable importance in projection (VIP) was obtained from partial least-squares discriminant analysis (PLS-DA). Principal Component Analysis (PCA), PLS-DA, VIP score, loading plot, correlation analysis, and classification of each metabolite with the corresponding metabolic pathway, were carried out using MetaboAnalyst 5.^1^ Statistical analyses were performed by the statistical multipackages of R software version 4.0.3 (R Core Team, 2020). Plots were displayed with Graphpad Prism version 5.01 (Graphpad Software Inc.) and the data visualisation package *Ggplot2* for the statistical programming language R.

## Results

**FIGURE 2:**
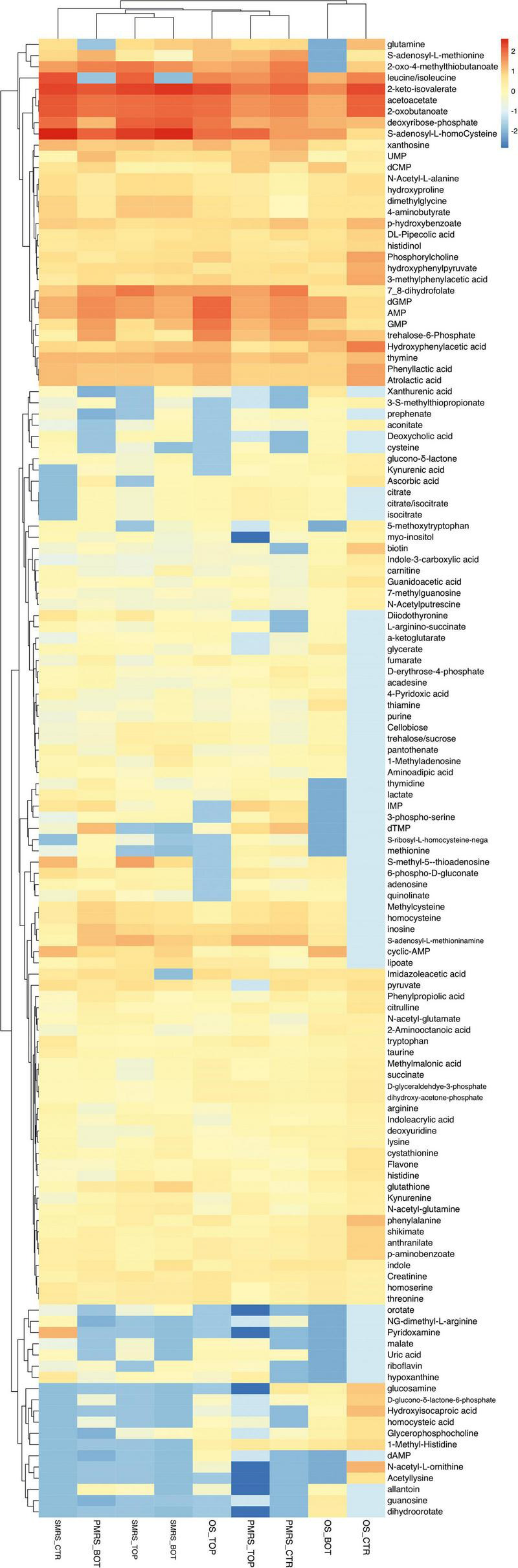
Heatmap showing unsupervised clustering based on the differential metabolites of importance derived from the pre-filtering analysis. Rows: differential metabolites; Columns: samples. The colours (red to cyano) represent the intensity changes of each metabolite relative to the mean control level. (MEA, malt extract agar; OS, original sandstone; OS Ctr, fungal colonies growth on MEA mixed with OS kept in the dark for the same time duration as the samples exposed to SVTs at room temperature; OS Top, fungal colonies growth on MEA mixed with OS accommodated in the upper part of the payload exposed to simulated space conditions; OS Bot, fungal colonies growth on MEA mixed with OS accommodated in the bottom part of the payload exposed to simulated space conditions; P-MRS, Phyllosilicatic Mars Regolith Simulant analogue; P-MRS Ctr, fungal colonies growth on MEA mixed with P-MRS kept in the dark for the same time duration as the samples exposed to SVTs at room temperature; P-MRS Top, fungal colonies growth on MEA mixed with P-MRS accommodated in the upper part of the payload exposed to simulated Mars conditions; P-MRS Bot, fungal colonies growth on MEA mixed with P-MRS accommodated in the bottom part of the payload exposed to Mars-like condition; S-MRS, Sulfate Mars Regolith Simulant analogue; S-MRS Ctr, fungal colonies growth on MEA mixed with S-MRS kept in the dark for the same time duration as the samples exposed to SVTs at room temperature; S-MRS Top, fungal colonies growth on MEA mixed with S-MRS accommodated in the upper part of the payload exposed to simulated Mars conditions; S-MRS Bot, fungal colonies growth on MEA mixed with S-MRS accommodated in the bottom part of the payload exposed to simulated Mars conditions).

Original sandstone, P-MRS, and S-MRS samples were analysed and compared to negative Ctr, to distinguish biomolecules derived from the cultivation media and from the fungus. The metabolomic analysis revealed 133 metabolites, classified as exclusively synthesized by the fungus (Figure 2).

### General Metabolic Profiling of *Cryomyces antarcticus* Exposed to Simulated Martian and Space Conditions

After pre-filtering analysis, the emerging 133 metabolites synthesized exclusively by the fungus were investigated. Hierarchical clustering heatmap analysis was performed to elucidate the characteristic patterns of expression of each metabolite. In Figure 2, the colours (red to cyano) represent the intensity changes of each metabolite relative to the mean control level. Individual samples (horizontal axis) and compounds (vertical axis) were separated using Hierarchical Clustering (Ward’s algorithm), with the dendrogram being scaled to represent the distance between each branch (distance measure: Pearson’s correlation). From Hierarchical Clustering, OS Top samples cluster with samples grown on Martian regoliths and particularly show great similarity with P-MRS samples (Figure 2).

**FIGURE 3:**
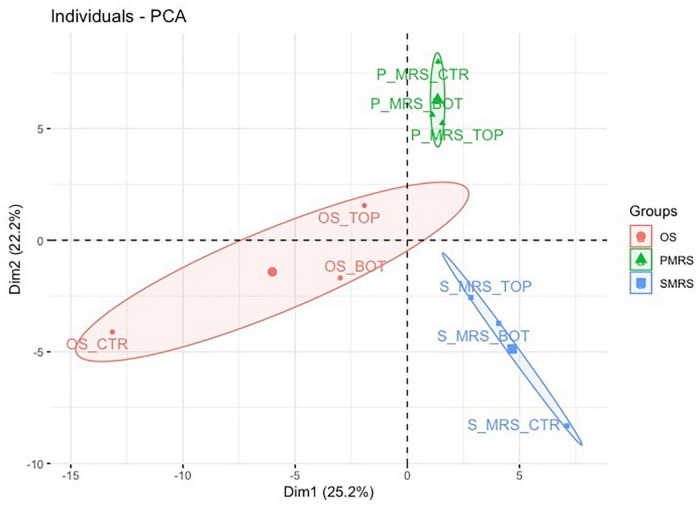
PCA score and loading plot for the first and second principal components considering all tested samples. (MEA, malt extract agar; OS, original sandstone; OS Ctr, fungal colonies growth on MEA mixed with OS kept in the dark for the same time duration as the samples exposed to SVTs at room temperature; OS Top, fungal colonies growth on MEA mixed with OS accommodated in the upper part of the payload exposed to simulated space conditions; OS Bot, fungal colonies growth on MEA mixed with OS accommodated in the bottom part of the payload exposed to simulated space conditions; P-MRS, Phyllosilicatic Mars Regolith Simulant analogue; P-MRS Ctr, fungal colonies growth on MEA mixed with P-MRS kept in the dark for the same time duration as the samples exposed to SVTs at room temperature; P-MRS Top, fungal colonies growth on MEA mixed with P-MRS accommodated in the upper part of the payload exposed to simulated Mars conditions; P-MRS Bot, fungal colonies growth on MEA mixed with P-MRS accommodated in the bottom part of the payload exposed to Mars-like condition; S-MRS, Sulfate Mars Regolith Simulant analogue; S-MRS Ctr, fungal colonies growth on MEA mixed with S-MRS kept in the dark for the same time duration as the samples exposed to SVTs at room temperature; S-MRS Top, fungal colonies growth on MEA mixed with S-MRS accommodated in the upper part of the payload exposed to simulated Mars conditions; S-MRS Bot, fungal colonies growth on MEA mixed with S-MRS accommodated in the bottom part of the payload exposed to simulated Mars conditions).

PCA was used for unsupervised variation analysis to detect groups and identify thediscriminating metabolites among selected sample groups (Figure 3).

The resulting total variance from the PCA is 47.4%, in which the contribution of Dimension 1 (Dim1) is 25.2% while that of Dimension 2 (Dim2) is 22.2% (Figure 3).

The result of Dim1 and Dim2 clearly showed how data sets corresponding to the same cultivation medium (e.g., OS Ctr, OS Bot, and OS Top) are characterised by greater similarity, forming three larger and specific clusters (e.g., OS, P-MRS, and S-MRS) based on the nature of the added mixture of minerals. Each cluster (OS, P-MRS, and S-MRS) is distinct and separate from each other (Figure 3). Moreover, it is clearly evident that OS, P-MRS, and S-MRS Ctr samples are those with the greatest variance (Figure 3). Instead, samples that were directly exposed to simulated space (OS Top) and Martian conditions (P-MRS and S-MRS Top) show a more similar tendency (Figure 3).

### Metabolic Profiling of Control Samples Grown on Different Substrata

**FIGURE 4:**
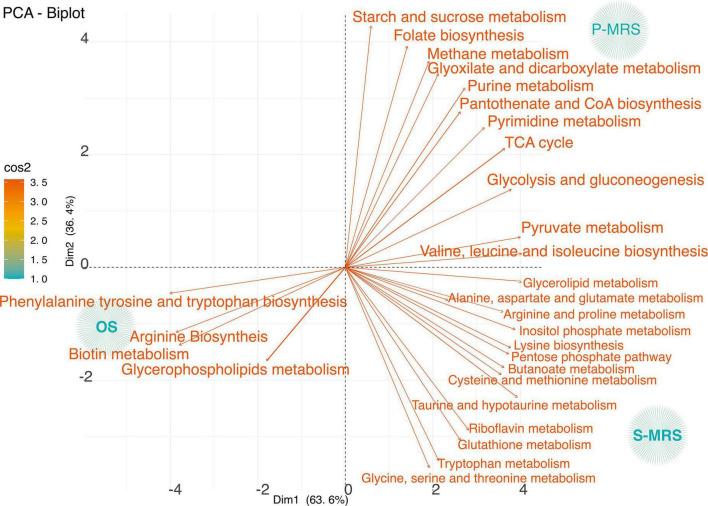
PCA loadings plot of all measured primary metabolite profiles for three controls samples classified according to the metabolic pathways considering only control samples – those kept in the dark for the same time duration as the samples in simulated conditions at room temperature. The quality of representation of the variables on the factor map is given by cos2 (square cosine, squared coordinates). The colour (red to orange) indicates high cos2 values meaning a good representation of the variable on the principal component. (MEA, malt extract agar; OS, original sandstone; P-MRS, Phyllosilicatic Mars Regolith Simulant analogue; S-MRS, Sulfate Mars Regolith Simulant analogue).

To analyse our first purpose, how the different regolith affected *C. antarcticus*, we analysed only the control samples without exposure to SVT treatments. Figure 4 reports the correlation degree of OS, P-MRS, and S-MRS Ctr, investigating in detail the main metabolites that are responsible for the differences among those samples.

The variance among OS, P-MRS, and S-MRS Ctr in relation to the identified pathways was investigated. Based on *p-value* and impact values, metabolites belonging to the pathways of arginine biosynthesis, phenylalanine, tyrosine, and tryptophan biosynthesis, and biotin metabolism were the most affected in OS Ctr (Figure 4). On the contrary, metabolites found in the P-MRS Ctr, a group in glyoxylate and dicarboxylate metabolism, purine and pyrimidine metabolism, TCA cycle, and pyruvate metabolism (Figure 4). Finally, the most abundant metabolites in the S-MRS Ctr classify into taurine and hypotaurine metabolism, glutathione metabolism, alanine, aspartate and glutamate metabolism, cysteine and methionine metabolism, riboflavin metabolism, and tryptophan metabolism (Figure 4).

**FIGURE 5:**
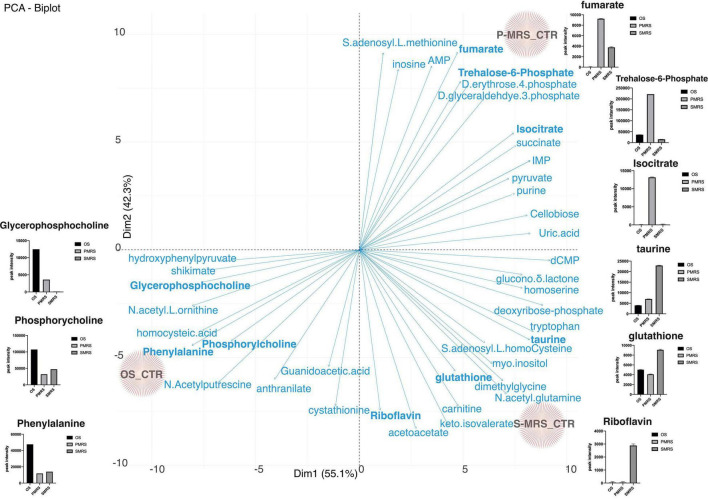
PCA loadings plot of all measured primary metabolite profiles for the three controls (OS Ctr; P-MRS Ctr; S-MRS Ctr). Differences in the content of peculiar metabolites for each substrate are reported through the boxplot. [MEA, malt extract agar; OS, Original Antarctic Substratum (black square); OS Ctr, fungal colonies growth on MEA mixed with OS kept in the dark for the same time duration as the samples exposed to simulated space conditions at room temperature; P-MRS, Phyllosilicatic Mars Regolith Simulant analogue (light grey square); P-MRS Ctr, fungal colonies growth on MEA mixed with P-MRS kept in the dark for the same time duration as the samples exposed to simulated Martian condition at room temperature; S-MRS, Phyllosilicatic Mars Regolith Simulant analogue (dark grey square); S-MRS Ctr, fungal colonies growth on MEA mixed with S-MRS kept in the dark for the same time duration as the samples exposed to simulated Martian condition at room temperature].

The differentiation of each Ctr sample is peculiar and is determined by metabolites that are significantly present. Figure 5 shows the PCA biplot derived from metabolites identified from OS, P-MRS, and S-MRS Ctr. Arrows represent metabolites responsible for the divergence among eachtreatment. For each metabolite with a significance *p* < 0.05, we have reported a box-plot. For instance, P-MRS Ctr is characterised by a high peak intensity value of trehalose-6-phosphate, isocitrate, and fumarate. In contrast, S-MRS Ctr is characterised by a high relative abundance of riboflavin, glutathione, and taurine (Figure 5). Finally, OS Ctr shows high peak intensity values of phenylalanine, phosphorylcholine, and glycerophosphocholine (Figure 5).

### Effect of Exposure to Simulated Martian and Space Conditions on Metabolites Stability

To verify the second purpose of our work, we determined the common relative behaviour in all samples, considering the different cultivation mediums (OS substratum, P-MRS, and S-MRS analogues) as well as the three different exposure conditions (Ctr, Bot, and Top).

**FIGURE 6:**
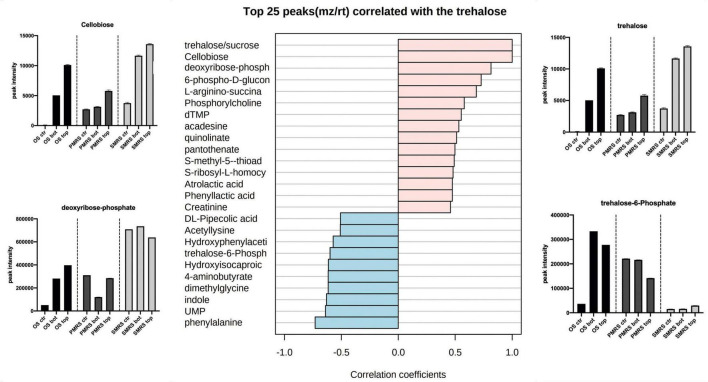
The top 25 metabolites associated with carbohydrates were considered as osmolytes. Pearson correlation was used as a distance measure (colour pink: positive correlation, colour cyano: negative correlation, within a vertical bar plot). Differences in the content of peculiar positive and negative correlated metabolites into different samples are reported through the boxplot. (MEA, malt extract agar; OS, original sandstone; OS Ctr, fungal colonies growth on MEA mixed with OS kept in the dark for the same time duration as the samples exposed to SVTs at room temperature; OS Top, fungal colonies growth on MEA mixed with OS accommodated in the upper part of the payload exposed to simulated space conditions; OS Bot, fungal colonies growth on MEA mixed with OS accommodated in the bottom part of the payload exposed to simulated space conditions; P-MRS, Phyllosilicatic Mars Regolith Simulant analogue; P-MRS Ctr, fungal colonies growth on MEA mixed with P-MRS kept in the dark for the same time duration as the samples exposed to SVTs at room temperature; P-MRS Top, fungal colonies growth on MEA mixed with P-MRS accommodated in the upper part of the payload exposed to simulated Mars conditions; P-MRS Bot, fungal colonies growth on MEA mixed with P-MRS accommodated in the bottom part of the payload exposed to Mars-like condition; S-MRS, Sulfate Mars Regolith Simulant analogue; S-MRS Ctr, fungal colonies growth on MEA mixed with S-MRS kept in the dark for the same time duration as the samples exposed to SVTs at room temperature; S-MRS Top, fungal colonies growth on MEA mixed with S-MRS accommodated in the upper part of the payload exposed to simulated Mars conditions; S-MRS Bot, fungal colonies growth on MEA mixed with S-MRS accommodated in the bottom part of the payload exposed to simulated Mars conditions).

**FIGURE 7:**
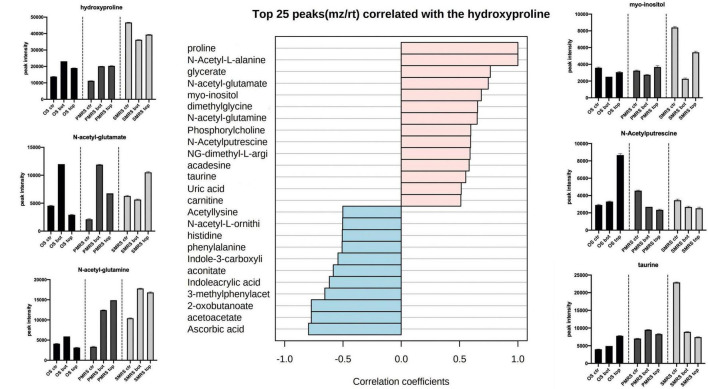
Top 25 metabolites associated with *N*-methyl, *N*-acetyl-substituted,and derived amino acids were considered as osmolytes. Pearson correlation was used as a distance measure (colour pink: positive correlation, colour cyano: negative correlation, within a vertical bar plot). Differences in the content of peculiar positive correlated metabolites into different samples are reported throughthe boxplot. (MEA, malt extract agar; OS, original sandstone; OS Ctr, fungal colonies growth on MEA mixed with OS kept in the dark for the same time duration as the samples exposed to SVTs at room temperature; OS Top, fungal colonies growth on MEA mixed with OS accommodated in the upper part of the payload exposed to simulated space conditions; OS Bot, fungal colonies growth on MEA mixed with OS accommodated in the bottom part of the payload exposed to simulated space conditions; P-MRS, Phyllosilicatic Mars Regolith Simulant analogue; P-MRS Ctr, fungal colonies growth on MEA mixed with P-MRS kept in the dark for the same time duration as the samples exposed to SVTs at room temperature; P-MRS Top, fungal colonies growth on MEA mixed with P-MRS accommodated in the upper part of the payload exposed to simulated Mars conditions; P-MRS Bot, fungal colonies growth on MEA mixed with P-MRS accommodated in the bottom part of the payload exposed to Mars-like condition; S-MRS, Sulfate Mars Regolith Simulant analogue; S-MRS Ctr, fungal colonies growth on MEA mixed with S-MRS kept in the dark for the same time duration as the samples exposed to SVTs at room temperature; S-MRS Top, fungal colonies growth on MEA mixed with S-MRS accommodated in the upper part of the payload exposed to simulated Mars conditions; S-MRS Bot, fungal colonies growth on MEA mixed with S-MRS accommodated in the bottom part of the payload exposed to simulated Mars conditions).

Correlation analysis was performed to explore the same pattern of change between the metabolites. We identified two main correlation patterns among the metabolites investigated. The first pattern is related to carbohydrates (trehalose; Figure 6), the second one is connected to a specific group ofsubstituted and derived amino acids (hydroxyproline; Figure 7). Metabolites showingthe same relative attitude are reported in Figure 5. To carry out this analysis we considered trehalose phosphate as a metabolite that exhibits an increase in all Top conditions, and we investigated which metabolites correlate with it (only pink colour bar, Figures 6, 7).

As reported in Figure 6, trehalose shows a strong positive correlation with cellobiose (*r* = 1), deoxyribose-phosphate (*r* = 0.81) and 6-phospho-D-gluconolactone (*r* = 0.72). On the contrary, the correlation with phenylalanine (*r* = −0.73), UMP (*r* = −0.63)and particularly with trehalose-6-phosphate (*r* = −0.59) results to be strongly negative. The concentration of trehalose, cellobiose, and deoxyribose-phosphate is higher in S-MRS samples compared to OS and P-MRS samples. In addition, the highest signal intensity corresponds to S-MRS Top and Bot, which are the ones exposed to simulated Mars conditions.

Figure 7 shows the pattern correlation related to *N*-methyl, *N*-acetyl-substituted, and derived amino acids. Proline, a functional compound in fungi, showed to have high positive correlation with several *N*-acetyl amino acidic compounds, *N*-acetyl-L-alanine (*r* = 1), *N*-acetyl-glutamate (*r* = 0.76), *N*-acetyl-glutamine (*r* = 0.65), *N*-acetylputrescine (*r* = 0.59), and other organic acids, such as phosphorylcholine (*r* = 0.59) and carnitine (*r* = 0.51). Hydroxyproline shows positive correlation also with sulfonate solutes, taurine (*r* = 0.58) and uric acid (*r* = 0.51). High negative pattern correlation is shown towards ascorbic acid (*r* = −0.79), acetoacetate (*r* = −0.77) and 2-oxobutanoate (*r* = −0.77). Instead, *N*-acetyl-L-ornithine shows a negative correlation with the groups of *N*-acetyl amino acidic compounds mentioned earlier. In addition, the highest proline values were found in S-MRS samples. Moreover, *N*-acetyl-glutamine and *N*-acetyl-glutamate show high peak intensity also in P-MRS samples. Instead, the highest signal of *N*-acetyl-ornithine, compared to other *N*-acetyl compounds, results in OS Top, while the peak intensity signal inP-MRS and S-MRS samples shows the same trend.

## Discussion

One of the main goals of future exploration missions on Mars and other planetary bodies of our solar systemwill be the detection of possible traces of present or past life. However, the conditions experienced by the molecules in space or on extraterrestrial planetary surfaces (e.g., Mars) are different from those experienced on Earth’s surface. The interpretation of the data collected during the ongoing and future space robotic missions might therefore be misinterpreted. For these reasons, the study of cells forming complex biomolecules’ behaviour under these conditions is an essential step of astrobiological investigation. In the frame of ground-based simulations (SVTs) of the BIOMEX project, we investigated the endurance of fungal biomolecules after the exposure of the extremophilic fungus *C. antarcticus* to simulated Martian and space conditions, within the EXPOSE-R2 facility. Here, for the first time, we investigated the stability/degradation of biomolecules through a -omic approach, since mass spectrometers is one of the instruments onboard the European Rosalind Franklin rover, allowing the analysis of small organics that might be indicative of extant or extinct compounds related to life as we know it (Goesmann et al., 2017).

Results derived from metabolomics analysis provided a global snapshot of all metabolites synthetized by the fungal colonies during growth and dehydration on Martian regolith analogues and on Antarctic sandstones. Moreover, a selected pool of molecules typically synthetized by the fungus emerged to be stable in its cellular environment even after the exposure to simulated Martian and space conditions. In particular, our results suggest that, among the molecular targets to be focused on during the future life-detection missions, those belonging to the chemical class of osmolytes, especially trehalose and cellobiose or membrane metabolites – glycerophosphocholine, choline, and myo-inositol – should be considered as suitable biosignature candidates. Metabolomics allows us to identify all the metabolomic components highlighting how this technique is a valid approach either to evaluate the stability of the biomolecules under simulated Martian and space conditions or to study if the presence of Martian regolith analogues may affect the fungal metabolism, during its growth before dehydration.

### Effects of the Interactions With Martian Mineral Analogues

The first project’s aim was to investigate and test whether the metabolome of *C. antarcticus* could differ if the fungus has been grown and dehydrated on two Martian regolith analogues compared to colonies grown on Antarctic sandstones. On a molecular scale, results of metabolomic measurements indicated that metabolites change dramatically not only compared to OS samples but differ also between samples grown on the two different Martian regolith analogues (Figure 4). Over-expressed metabolites detected in both P-MRS and S-MRS samples are typically associated with a response to water and osmotic stress conditions. Indeed, the over-expressed metabolites belong to specific pathways involved in glycerol accumulation, storage of compatible sugars, organic acids, and other specific molecules (e.g., glutathione, riboflavin, myo-inositol, *N*-acetyl-putrescine, and *N*-acetyl-ornithine). All of the metabolites mentioned above are typically involved in stress resistance as adaptative strategies used by fungi to overcome extreme conditions (Williamson et al., 2002; Brown et al., 2007; Valdés-Santiago and Ruiz-Herrera, 2014).

### The Exposure Effects of Fungal Colonies Grown on P-MRS Analogue

Among the over-expressed metabolites identified in P-MRS Ctr, isocitrate, fumarate, and succinate are those with the highest peak intensity values (Figure 5). These metabolites are involved in the tricarboxylic acid cycle (TCA) and in the glyoxylate cycles (Figure 4). The latter pathway could be utilised to bypass the CO_2_-generating steps of the TCA cycle and allow the net assimilation of carbon from C_2_ compounds, allowing microorganisms to replenish the pool of TCA cycle intermediates necessary for gluconeogenesis and other biosynthetic processes. The result of the glyoxylate cycle is the production of succinate from two molecules of acetyl-CoA for the synthesis of carbohydrates, such as trehalose (Barrett et al., 1970; Eastmond and Graham, 2003; Kondrashov et al., 2006). There is also evidence supporting that the glyoxylate cycle may allow the growth of halophilic Archaea in hypersaline lakes (Oren and Gurevich, 1994). Indeed, glyoxylate cycle links catabolic activities to biosynthetic capacities, allowing cells to use fatty acids or C_2_-units like ethanol or acetate as their sole carbon source. Acetate may play a role in the nutrition of natural communities of halophilic Archaea, as it is produced from glycerol (the main carbon source in nature) in hypersaline lakes by some species of halophiles (Oren, 2017). The activities of glyoxylate cycle key enzymes – malate synthase and isocitrate lyase – are required to synthesise carbohydrate precursors only when the carbon source is acetate, not when the carbon source is a compound with three or more carbons, such as lactate. The activities of isocitrate lyase and malate synthase in cell extracts of *Haloferax volcanii*, a halophilic archaeon capable of growing in minimal medium with acetate as the only carbon source, revealed no activity when lactate was the carbon source. On the contrary, when acetate was the sole carbon source, the activity of both the enzymes was high (Serrano et al., 1998; Kuprat et al., 2020). *Cryptococcus neoformans* pulmonary infection studies revealed the expression of genes encoding glyoxylate pathway functions (e.g., isocitrate lyase) – those required for growth on acetate – for full virulence. Furthermore, transcripts for transporters (such as monosaccharides, iron, copper, and acetate) and stress-response proteins were increased, indicating a nutrient-limited and hostile host environment (Hu et al., 2008). It could be assumed that also the dehydration process may lead to osmotic and oxidative stress and the activation of this alternative pathway that has also been found in other fungi (Lorenz and Fink, 2001; Kunze et al., 2006).

The over-expression of folate biosynthesis is associated with purine and pyrimidine metabolism. Purine and pyrimidine nucleotides are major energy carriers, subunits of nucleic acids, and precursors for the synthesis of nucleotide cofactors, such as nicotinamide adenine dinucleotide (NAD) and S-adenosylmethionine (SAM). It is interesting to observe that SAM was found in high concentration (data not shown) in a Martian regolith analogue cultivation medium, where the mineral mixtures composition is low of nitrogen and sulfur compared with the S-MRS cultivation medium. Indeed, previous studies performed on *Saccharomyces cerevisiae* showed that the accumulation of SAM in the vacuoles is necessary for yeast growth under sulfur and nitrogen starvation and for the balance of the homeostasis of sulfur amino acids (Shobayashi et al., 2007). These results were confirmed by the detection of S-methyl-cysteine, homocysteine, and cysteine in P-MRS samples. These endogenous sulfur-containing amino acids are known to give to the microorganism osmotic tolerance under dehydration stress conditions (Oren, 2008). Moreover, SAM is known to be related to ergosterol synthesis and to act as fungal cell-membrane constituents which may play fundamental functions to maintain membrane fluidity and permeability. As reported for *S. cerevisiae* as well as on other halotolerant yeasts (*Zygosaccharomyces rouxii, Debaryomyces hansenii, Candida membranefaciens*, and *Yarrowia lipolytica)*, salt stress and dehydration affect the lipids composition with an increasing amount of ergosterol (Turk et al., 2007).

Regeneration and accumulation of glucose provide the necessary elements for the synthesis of trehalose. Trehalose, a non-reducing disaccharide consisting of two units of glucose (α-D-glucopyranosyl-1,1-α-D-glucopyranoside) is widespread in a variety of organisms: bacteria, yeast, fungi, lower and higher plants, as well as insects and other invertebrates (Elbein et al., 2003); and plays the role of energy source, osmolyte or protein/membrane protectant. Trehalose is accumulated in anhydrobiotic organisms to survive the complete dehydration (Drennan et al., 1993), by preserving the membranes during drought periods (Crowe et al., 1984). As suggested in previous works anhydrobiosis provide protection against extreme environmental conditions (Billi et al., 2000; Kranner et al., 2008). In yeast, trehalose plays a role in osmotic stress (Hounsa et al., 1998), temperature, and desiccation tolerance (Hottiger et al., 1987). However, the high levels of trehalose could be the result of a direct response to the dehydration process to which the fungus was subjected before treatments, rather than a response against the alkalophilic Martian regolith analogue. The high concentration of trehalose detected in the P-MRS samples, suggests that the cultivation medium may affect and stimulate the fungus to produce a large amount of trehalose, especially when compared with OS and S-MRS samples, which show a minor production of metabolites involved in those pathways necessary for the cell to accumulate glucose and then synthesise trehalose.

### The Exposure Effects of Fungal Colonies Grown on S-MRS Analogue

Sulfate Mars Regolith Simulant analogue-Ctr shows an over-expression of the metabolites typically synthesized by the cells in pathways against oxidative stress. The high levels of 6-phospho-D-gluconate, D-gluconolactone-6-phosphate, and deoxyribose-phosphate, detected in S-MRS samples, are part of the pentose phosphate pathway (PPP; Figures 4, 5). PPP is known to be important for generating NADPH, which is a source of reducing energy, as well as a variety of sugar molecules that are required for the biosynthesis of nucleic acids and amino acids (Miosga and Zimmermann, 1996; Slekar et al., 1996; Kondo et al., 2004). The ability of a microorganism to adapt to external stressors probably depends on its ability to maintain metabolic homeostasis. As shown in *C. neoformans*, among the metabolic pathways upregulated under stressed conditions, PPP is important for protecting the fungus from oxidative stress (Hu et al., 2008; Upadhya et al., 2013). The reducing equivalent NADPH produced by the PPP is essential for the maintenance of functional glutathione and thioredoxin-dependent enzymes systems to defend cells against oxidative damages (Minard and McAlister-Henn, 2005; Brown et al., 2007). Reactive Oxygen Species (ROS) can cause irreversible damages to cellular components and thus are normally rapidly detoxified by antioxidant defence systems including enzymes, such as catalases, superoxide dismutase, small proteins (thioredoxin and glutaredoxin), and antioxidant molecules, such as the glutathione (Cabiscol et al., 2000). These mechanisms involve the glutathione redox system, which is well known to be present also in black fungi (Jürgensen et al., 2001). Under stress conditions, glutathione reduced (GSH) is utilised to scavenge the ROS directly by donating a reducing equivalent (H^+^ + e^–^) from its thiol group of cysteine to other unstable ROS (Han et al., 2017). High levels of glutathione detected in the S-MRS samples, therefore, suggest a sustained and efficient ROS scavenging response of the fungus. Among the metabolites detected in S-MRS Ctr, riboflavin is one of those with the highest peak abundance values (Figure 5). The fungus may host other antioxidant defence molecules to protect itself, which includes the vitamin riboflavin which is also an antioxidant and free radical scavenger (Durusoy et al., 2002).

Riboflavin is the precursor for the coenzymes flavin mononucleotide (FMN) and flavin adenine dinucleotide (FAD), which participate in various oxidation and reduction reactions in the cells (Burgess et al., 2009). Glutathione reductase requires FAD as a cofactor along with NADPH for the reduction of oxidised glutathione to GSH (Masella et al., 2005). Thus, riboflavin overproduction by *C. antarcticus* could enhance the GSH metabolism in response to oxidative stress. These suggest that the more acidic S-MRS regolith analogue may induce more endogenous production of ROS. The idea is also supported by the high levels of taurine found in the S-MRS Ctr samples (Figure 5). Indeed, in a previous metabolomic and genomics study performed on *Acidomyces richmondensis* isolated from Acid Mine Drainage (AMD) biofilm, its genome revealed a gene that was involved in the biosynthesis of taurine metabolites (Mosier et al., 2016). When exposed to environmental stressors, taurine is involved in several physiological roles, such as protecting proteins, nucleic acids, and membranes against ROS (Andres and Bertin, 2016).

### The Exposure Effects of Fungal Colonies Grown on OS Substratum

Aromatic amino acids, such as tyrosine, tryptophan, and phenylalanine, as well as basic amino acid arginine, were those with the highest peak intensity values in OS samples (Figures 4, 5).

Amino acids are biologically important organic compounds and play fundamental roles in a multitude of functions, including protein synthesis, cell growth, development, and production of energy. Arginine, for instance, plays important roles in a multitude of processes, including protein synthesis, signal transduction, osmotic pressure homeostasis, and cell growth (Bedford and Richard, 2005; Wu et al., 2009). Indeed, arginine is one of the most versatile amino acids and its biosynthetic pathways are essential as a precursor for the synthesis of different chemical groups of molecules, including polyamines. Polyamines are recognised to act in cell growth and developmental processes, and aromatic amino acids are also known to improve cell growth as already reported for the Antarctic basidiomycetous yeast *Mrakia blollopis* (Tsuji, 2016). However, as reported for P-MRS and S-MRS Ctr, also in OS Ctr the dehydration process, might be a source of osmotic and oxidative stress. During an abiotic event, such as salinisation, freezing, or dehydration, microorganisms, are subjected to hyperosmotic stress (Yancey et al., 1982). An increase in the external osmolarity produces a rapid efflux of water from the cell to the surrounding environment. This implied a decrease in the cellular volume and turgor pressure and may result in cellular death (Chamberlin and Strange, 1989). A common biological mechanism to counteract hyperosmotic stress involves the cellular accumulation of “compatible solutes” or osmolytes (Brown and Simpson, 1972; Yancey et al., 1982). High levels of glycerophosphocholine (GPC), which is involved in the glycerophospholipids metabolism (Figures 4, 5), is a direct response of the organism to overcome the hyperosmotic stress caused by the dehydration process. GPC is thought to stabilise membranes, as well as protein structure and function during osmotic stress (Kwon et al., 1995; Popova and Busheva, 2001). Indeed, it was already shown in *S. cerevisiae* that osmotic stress induces a rapid turnover of the membrane lipids to produce a soluble methylamine product, GPC, which works as an osmoprotectant and even as an enhancer of cell growth in yeast during hypersaline and dehydration stress (Kwon et al., 1995; Kiewietdejonge et al., 2006).

### Stability of Fungal Osmolyte Under Simulated Martian and Space Conditions

Metabolomics analysis revealed that OS, P-MRS, and S-MRS samples differ dramatically according to the type of growth media (Figure 3). However, differences between the samples may derive not only from the cultivation medium but also from the type of experimental condition (OS – space-like conditions; P-MRS and S-MRS – Mars-like conditions). In Figures 2, 3, OS, P-MRS, and S-MRS Top samples show a great similarity, having a closer position to each other along the two dimensions of the PCA. It may be justified because OS, P-MRS, and S-MRS Top are accommodated in the top part of the hardware and are subjected to additional parameters during the test. Indeed, UV irradiation may have contributed to altering the metabolites in the same way regardless of the media composition. However, the analysis clearly showed that there is a pool of metabolites that are stable, and therefore detectable, even after treatment (Figures 4, 5). These molecules belong to a chemical compound known as an osmolyte. Amino acids and derivatives, polyols, sugars and derivatives, methylamines, and methyl-sulfonium compounds are considered osmolytes (Kempf and Bremer, 1998; Wood et al., 2001; Yancey, 2005; Essendoubi et al., 2007; Empadinhas and Da Costa, 2008). Among the osmolytes, we focused on trehalose since it was one of the most abundant in all the samples and it is known to have a cell-protective role in black fungi (Onofri et al., 1999; Zakharova et al., 2013, 2014). As shown in Figure 6, analyzing the signal pattern in the nine different samples, trehalose shows a high correlation with another non-charged disaccharide, cellobiose, which has previously been found as an osmolyte in Archaea (Zancan and Sola-Penna, 2005). Instead, a strong negative correlation was found with trehalose-6-phosphate (Tre-6-P; Figure 6). OS, P-MRS, and S-MRS Top samples show an increase in the abundance of trehalose compared to those detected for Tre-6-P. It is particularly evident in S-MRS samples. Accumulation of trehalose to the detriment of Tre-6-P may be the result of direct exposure of Top samples to UV irradiation which acted to remove the γ-phosphate group as already observed for the slow UV-degradation of ATP in simulated Martian environments (Schuerger et al., 2008). On Mars, exposure to surface UV radiation can degrade common biologically relevant organic molecules, and lead to misinterpreted results. Rocks and minerals can provide a shield against UV (Carrier et al., 2019). Moreover, as suggested by studies on the desert cyanobacterium *Chroococcidiopsis*, the accumulation of two specific sugars, trehalose, and sucrose, could not only increase the chance of cell survival in biofilms dried for 7 years mixed with P-MRS analogue and exposed to a Mars-like UV flux but also allow a slowing down in the rate of degradation of biosignatures embedded in planetary mineral analogues (Fagliarone et al., 2020). In addition, studies performed on the black yeast-like fungus *Aureobasidium subglaciale* showed a correlation among the resistance to γ-radiation, UV light, and heavy metals ions, and the accumulation of trehalose (Liu et al., 2017). However, trehalose is also highly correlated in the signal pattern relative to deoxyribose-phosphate, a biomarker of DNA lesion after radical and ROS attacks caused by endogenous or exogenous oxidizing agents, such as UV radiation (Valavanidis et al., 2006). Surprisingly, the highest levels of deoxyribose-phosphate were found in S-MRS samples (Top, Bot, and Ctr; Figure 6), confirming an active oxidative stress response already emerged in S-MRS Ctr (Figures 3, 4). As discussed for trehalose, we focused on additional compounds with similar functions and widespread fungal occurrences, including betaine and proline (Takagi, 2008; Figure 7). Proline shows a high positive correlation with *N*-acetyl-glutamate and *N*-acetyl-glutamine which are osmolytes and were found in *Halolactibacillus halophilus* to be precursors of *N*-acetyl-ornithine. The latter as well as acetyl-lysine are considered osmoprotective molecules (Lai et al., 1991, 1999, 2000). For both no peak intensity signal was detected (data not shown) resulting in a negative correlation with proline and other *N*-acetyl subsisted amino acids. Proline is positively correlated with other organic osmolytes, especially myo-inositol and taurine, known to maintain a regular cell volume during osmotic disturbances in mammalian cells (Law, 1994; Weik et al., 1998) and essential in all eukaryotes, including pathogenic fungi and protozoa, as well as eubacterial pathogens, including mycobacteria (Michell, 2008).

Results provided from the current work can be merged to previous analyses performed on *C. antarcticus* in the context of SVTs of the BIOMEX experiment which revealed good viability after the SVT treatments. Indeed, the fungus demonstrated a good survivability percentage and metabolic activity recovery after SVTs treatments. In particular, fungal colonies grown on S-MRS analogue and exposed to Top conditions, show 57% of survivors (Pacelli et al., 2019). Results from current work suggest that during the dehydration process the fungus, regardless of the cultivation medium, is stimulated to synthesise osmocompatible molecules with a protective function. The great survivability reported in previous works (Pacelli et al., 2018, 2019) after the dehydration processes may be explained by the enhanced production of these molecules during the dehydration process. The high viability showed by *C. antarcticus* after SVTs treatments may be explained by the anhydrobiotic state and the presence of melanin in cell walls (Pacelli et al., 2019). During the anhydrobiotic state, organisms enter a reversible metabolic dormancy. In this condition, non-reducing sugars are accumulated in the cytoplasmatic environment, replacing water molecules, preventing membrane phase transition, and allowing cytoplasmic vitrification (Sun and Leopold, 1997; Crowe et al., 1998). Trehalose is one of the non-reducing sugars accumulated during this reversible metabolic dormancy. From our results, it emerged as one of the most abundant osmolyte synthetized by the test organism and even one of the most stable compounds after the treatments. Trehalose can acts to stabilise the membrane and even the proteins’ structure (Jain and Roy, 2009), playing a key role in the survivability of *C. antarcticus*.

Mass spectrometers have been employed as payload instruments for planetary exploration since the 1970s (Ren et al., 2018). However, due to the miniaturisation onboard the rovers, the mass spectrometer undergoes a relatively low detection sensitivity. In the next space exploration missions, payload instruments must have high analytical capabilities to avoid pitfalls or misinterpretations, especially in the search for extant or extinct life traces. In this context, different applications and versatility of mass spectrometers explain why they are considered successful tools as mission payload instruments for the *in situ* investigation of planetary bodies. With the new technological advances in space research, new high-resolution mass analysers are emerging. Among them, the mass analyser Orbitrap used in this work is a powerful tool due to ultrahigh mass resolving power up to m/Δm ≥ 1,000,000 (FWHM; Denisov et al., 2012) leading it to emerge as a viable candidate for the next spaceflight applications (Arevalo et al., 2020).

## Conclusion

The potential of metabolomics technique to detect biomolecules as potential biosignatures and to investigate their stability under simulated Martian and space conditions is successfully achieved. It is of great interest for the next life-detection missions since the detection of these biosignatures may be indicative of extant or extinct life on other planetary bodies. The present analytical technique allows us to investigate the effects of the interactions with two Martian regolith analogues and its original Antarctic substrates with fungal colonies. Although any fungal metabolism could be different in the conditions on Mars or in outer space, the search for biosignatures on other planetary bodies of our Solar System should begin with the identification of molecules that are diagnostic of metabolic pathways common on Earth since it is the only example of life as we know so far. In this context, the results of this work allow us to expand our knowledge on these specific pools of molecules. The findings show that changes in the type of growth medium have a significant impact on the basal metabolism of the fungal. The addition of Martian regolith analogues increases osmotic and oxidative stress in the test organism significantly. In response to these stressful conditions, the fungus produces a pool of molecules known as osmolytes, which not only act to stabilise the membrane (trehalose) but also directly participate in the scavenging of free radicals, which are one of the most damaging factors in the Martian radiative and space environment. Furthermore, these molecules were found to be extremely resistant when directly exposed to simulated Martian and space conditions. These findings, in particular, allow us to expand the current molecular targets that will be identified in future space missions in search of possible traces of present or past life.

## Data Availability Statement

The raw data supporting the conclusions of this article will be made available by the authors, without undue reservation.

## Author Contributions

SO, J-PV, ER, and CP designed the research. SO and AT conceived and designed the experiment. FG and PL performed the experiments. FG, PL, AC, and CP analysed data. SO, AT, FG, and PL drafted the article with inputs from all other authors. All authors approved the submitted version.

## Conflict of Interest

The authors declare that the research was conducted in the absence of any commercial or financial relationships that could be construed as a potential conflict of interest.

## Publisher’s Note

All claims expressed in this article are solely those of the authors and do not necessarily represent those of their affiliated organizations, or those of the publisher, the editors and the reviewers. Any product that may be evaluated in this article, or claim that may be made by its manufacturer, is not guaranteed or endorsed by the publisher.

## Footnotes

1
https://www.metaboanalyst.ca/
